# Suitability of ‘Guidelines for Screening of Prosthetic Candidates: Lower Limb’ for the Eastern Cape, South Africa: A qualitative study

**DOI:** 10.4102/sajp.v74i1.396

**Published:** 2018-01-30

**Authors:** Luphiwo L. Mduzana, Surona Visagie, Gubela Mji

**Affiliations:** 1 Department of Rehabilitation Medicine, Faculty of Health Sciences, Walter Sisulu University, South Africa; 2 Centre for Rehabilitation Studies, University of Stellenbosch, South Africa

## Abstract

**Background:**

Major lower limb amputation has a severe impact on functional mobility. Mobility can be salvaged with a prosthesis, but this is not always the best option. It is often difficult to decide whether to refer someone for a prosthesis or not. A prosthetic screening tool ‘Guidelines for Screening of Prosthetic Candidates: Lower Limb’ was developed and is used for prosthetic prescription in parts of the Western Cape province of South Africa.

**Objectives:**

This study aimed to explore the suitability of the tool ‘Guidelines for Screening of Prosthetic Candidates: Lower Limb’ for use in the Eastern Cape province of South Africa.

**Method:**

A qualitative study was conducted with conveniently sampled occupational therapists (OTs) (*n* = 10), physiotherapists (PTs) (*n* = 12) and prosthetists (*n* = 6) in government employment in the Buffalo City Metro Municipality. Participants were trained in the use of the tool and used it for four weeks with patients. Their experiences of the tool were assessed through three focus group discussions with emergent themes being identified during inductive data analysis.

**Findings:**

Participants indicated that the tool could assist with prosthetic prescription, goal setting, communication and teamwork. They thought that the tool was multidisciplinary in nature, comprehensive and practical. Findings showed a lack of teamwork in this study setting. Resistance to change and a lack of time might also hamper implementation of the tool.

**Conclusion:**

The tool can assist with managing the backlog for prostheses and to guide prosthetic prescription in the Eastern Cape province.

**Clinical implications:**

A prosthesis can help to salvage functional mobility after lower limb amputation. However, not all people who had above knee amputation manage to walk with a prosthesis. The tool reported on in this article provides information that can guide prosthetic prescription and rehabilitation goals.

## Introduction

The impact of a major lower limb amputation on an individual’s functional mobility can be life-altering (Marzen-Groller et al. 2008). While a prosthesis can improve mobility and independence (Marzen-Groller et al. 2008; Webster et al. 2012), prosthetic rehabilitation does not always guarantee functional mobility and might not be the most optimal mobility device after amputation (Schaffalitzky et al. 2012). In some instances, restoring mobility through a wheelchair or crutches may be of more benefit (Condie et al. 2011). The decision whether a person should be referred for a prosthesis is challenging because post-prosthetic outcomes must be predicted based on pre-prosthetic ability (Gailey 2006).

The decision to provide a prosthesis or not is usually based on clinical expertise (Gailey 2006; Schaffalitzky et al. 2011; Van der Linde et al. 2003). This can cause variations in prescription as well as over- and under-use of prosthetic care. The prescription process lacks transparency for consumers and funders (Schaffalitzky 2010; Van der Linde et al. 2003), so standardised clinical guidelines, or a tool predicting prosthetic use, may result in more consistent and efficient clinical practice (Van der Linde et al. 2003).

In order to be suitable for use in a specific setting, a tool must gather relevant, useful information. It must be valid, reliable, sensitive and specific. Finally, the tool must be feasible, that is, easy to administer, and not add a cost or time burden (Martin & Kettner 2009). Thus, in the case of this study, a suitable tool will assist service providers to identify prosthetic candidates with accuracy, while not taking long to complete or interpret.

The only tool used to predict prosthetic ambulation, based on pre-prosthetic assessment that could be identified through published work, is the Amputee Mobility Predictor© (AMP). While feasible, reliable and valid, the AMP was developed in a well-resourced setting (Gailey et al. 2002). Services, environments and prosthetic components (Andrysek 2010; Kam et al. 2015; Wyss et al. 2015) differ between well-resourced and poorer-resourced settings. In poorer-resourced settings, diverse natural environments, seasonal changes and climate extremes as well as occupations that require strenuous physical activity, such as farming, necessitate that prosthetic components enable function in physically demanding circumstances (Kam et al. 2015). Indeed, the simpler prosthetic components often used in developing countries (Kam et al. 2015) require higher levels of physical conditioning and strength to use, and do not support the functional requirements of strenuous activities (Andrysek 2010; Wyss et al. 2015).

Distances, a lack of transport, financial constraints and a shortage of healthcare hamper rehabilitation in poorly resourced settings (Kam et al. 2015). South African studies describe little (Fredericks & Visagie 2013; Godlwana, Stewart & Musenge 2015) or no rehabilitation post-amputation (Ennion & Rhoda 2016). Ennion and Rhoda (2016) found a lack of stump preparation, prosthetic waiting periods of three years and poor functional outcomes in KwaZulu-Natal. They ascribe this to staff shortages, a lack of resources and transport, poor teamwork and communication, barriers in the natural environment and cultural factors. These challenges can impact prosthetic mobility negatively.

The AMP assesses sitting, standing, balance and locomotion. The ability to balance on the remaining leg has a strong correlation with prosthetic ability and use (Raya et al. 2010; Schoppen et al. 2003), whereas the use of mobility-assistive devices before the amputation correlates with decreased prosthetic provision (Mundell et al. 2016) and function (Roffman, Buchanan & Allison 2014). However, aspects such as age (Davie-Smith et al. 2017; Mundell et al. 2016; Resnik & Borgia 2015; Schoppen et al. 2003; Yilmaz et al. 2016), cause of amputation (Mundell et al. 2016), comorbidities (Resnik & Borgia 2015), cognition (Mundell et al. 2016) as well as the condition of the remaining and residual limbs (Raya et al. 2010; Roberts et al. 2006) also influence prosthetic functioning.

Weakness in the extensor and abductor muscle groups of the residual and remaining limbs impacts prosthetic ability negatively (Raya et al. 2010; Roberts et al. 2006), as do flexion contractures (Roberts et al. 2006). The residual limb should be free of wounds, with healthy skin and firm soft tissue (Roberts et al. 2006). Challenges with the shape and skin condition of the residual limb are often experienced in developing countries (Kam et al. 2015), making these important factors to take into consideration during prosthetic prescription.

The AMP does not consider all clinical, personal and environmental aspects that might influence prosthetic use in a developing country like South Africa. In some settings in the Western Cape province of South Africa, a prosthetic screening tool ‘Guidelines for Screening of Prosthetic Candidates: Lower Limb’^1^ is used to ensure that suitable candidates are referred for prosthetic consideration (PGWC 2010). The tool was developed in South Africa and includes issues such as the cause of amputation, comorbidities, cognition, condition of the remaining and residual limbs as well as balance and locomotor abilities.

Anecdotal information shows that in the Eastern Cape province, individual service providers use their discretion to decide whether to refer a person for a prosthesis. This *ad hoc* practice might discriminate against some patients and might lead to unsuccessful prosthetic fitting. In addition, because of a backlog, in excess of 600 patients, waiting times are long. If the prosthesis is fitted after a long waiting period, muscle atrophy and physical deconditioning (Roffman et al. 2014) increase the need for rehabilitation and negatively impact prosthetic function (De Boer-Wilzing et al. 2011).

This study aimed to explore the suitability of the tool ‘Guidelines for Screening of Prosthetic Candidates: Lower Limb’ for use in the Eastern Cape province of South Africa.

## Development of ‘The Guidelines for Screening of Prosthetic Candidates: Lower Limb’

‘The Guidelines for Screening of Prosthetic Candidates: Lower Limb’ was based on evidence gathered by Bakkes (1999) in a cross-sectional survey to determine variables that had a statistically significant impact on functional prosthetic ambulation. Study participants included persons with an above knee amputation who received rehabilitation at a specialised rehabilitation unit in the Western Cape province (*n* = 46). She found that functional walking with a prosthesis is dependent on pre-prosthetic ability to walk with crutches as well as residual limb full extension and adduction range, and normal muscle strength. Stump length, shape and preparation as well as the person’s pre-morbid level of fitness and comorbidities also play a role (Bakkes 1999). These findings confirmed known associations and point towards predictive validity (Katzenellenbogen & Joubert 2007). The findings were used to develop draft guidelines to identify prosthetic candidates. The draft guidelines were used by service providers and further refined. During this process, face and content validity (Katzenellenbogen & Joubert 2007) were also ensured. Finally, the tool as used in this study was formulated.

In total, 29 variables that might impact prosthetic use are assessed by the tool as presented in Appendix 1. The tool is formatted in four columns. The first contains the aspect or variable to be assessed such as aetiology. The second, third and fourth are headed contraindication or poor prognosis, negative predictor and positive predictor, respectively. The assessor ticks the relevant column for each variable. The number of ticks per column is calculated and used to determine if the user is a good candidate, needs intervention or is not a prosthetic candidate as shown in the final row in Appendix 1. The tool has not been tested for criterion validity, reliability, sensitivity or specificity.

## Methodology

A qualitative study was undertaken where focus group discussions were used to explore professionals’ perceptions and opinions on the tool. In order to find a way of addressing the huge prosthetic backlog (> 600) and long prosthetic waiting times in the Eastern Cape province, the first author, an orthoptist or prosthetist in the study setting, explored prosthetic prescription guidelines. He learned about the tool, and spent a month at a facility in Cape Town where the rehabilitation team used the tool to guide prosthetic prescription. There he familiarised himself with the theoretical background and practical implementation of the tool. None of the other authors was involved in the development of the tool or had used it clinically.

### Study setting

The Eastern Cape province has three government-subsidised hospitals with prosthetic services that employ around 25 prosthetists. The healthcare facilities selected for this study refer patients to one of these, that is, the orthotic and prosthetic department at Frere hospital. The study was conducted in the Buffalo City Metro Municipality. Specific settings included Frere hospital (tertiary level of care), Cecilia Makiwane Hospital (secondary level of care) Empilweni and S.S Gida clinics (primary level of care). Frere hospital is located close to the East London central business district (an urban area). Cecilia Makiwane Hospital is situated in Mdantsane, a peri-urban area in East London. Empilweni clinic is situated in an informal settlement. S.S Gida is situated on the outskirts of East London and caters mainly for people in the surrounding rural areas.

### Study population, sampling and participants

Rehabilitation service providers to persons with lower limb amputations in the study hospitals and clinics formed the study population. These included orthopaedic surgeons (number not available), physiotherapists (PTs) (20), occupational therapists (OTs) (15), prosthetists (6) and professional nurses (number not available). Professionals, such as social workers, who are not directly involved with prosthetic prescription were excluded. The sampling process was one of convenience as members of the study population were asked to volunteer to participate in the study.

The professional nurses and orthopaedic surgeons declined participation. The professional nurses indicated that they do not play a role in prosthetic prescription and rehabilitation. A spokesperson for the orthopaedic surgeons explained that as the PTs and OTs decided which patients are prosthetic candidates, they would not have any use for the tool. He further explained that because of a high turnover amongst junior doctors, it would be difficult to ensure continuity in the use of the tool. The study was introduced to potential participants through information sessions by the first author as shown in Table 1. Ten OTs, 12 PTs and six prosthetists participated in the study (total *n* = 28). Participants received training in the use of the tool (Table 1). On completion of the training, each participant received 10 copies of the tool and was asked to use it with patients. Participants were free to make extra copies if needed, or use it with fewer than 10 patients if fewer opportunities were presented.

**TABLE 1:** An overview of study processes (created by author).

Activity	Purpose	Duration	Date	No. of times repeated
Information sessions	To introduce the study To identify study participants	1 h	April–May 2015	3
Training sessions	To train participants in the use of the tool	1 h	June 2015	3
Participants use tool	To allow participants to gain practical experience of the tool	1 month	July 2015	0
Focus groups	To explore participants’ opinion on the tool	30–45 min	August 2015	3

*Source*: Authors’ own work

### Data collection and analysis

Therapists from the clinics and Cecilia Makiwane Hospital joined their colleagues at Frere hospital for three focus group discussions (one per professional group). Data were collected in English, by the first author. The discussions were audio-recorded. Focus group discussions were guided by an interview schedule. The interview schedule was piloted with three people prior to the focus group discussions and included the following aspects:

- general thoughts on the tool
- usefulness of the tool
- whether the tool assisted in identifying prosthetic candidates
- how difficult or easy the tool was to use
- whether using the tool added to their work load
- whether the tool could be used in the Eastern Cape province.

Inductive analysis was performed according to the phases described by Braun and Clarke (2006), that is, familiarisation through transcribing and reading data, identification of codes, categorising of codes into possible themes, reviewing, defining and naming themes. The first two authors analysed the data separately after which themes were compared and consensus was reached.

### Authenticity

Credibility, transferability, consistency and neutrality were taken into consideration to determine authenticity in this study (Denzin & Lincoln 2005). These aspects were dealt with through triangulation firstly of three different sources of information and secondly by using multiple analysts as described by Patton (1999). Rival opinions were presented and explored (Patton 1999). Findings are supported through narrative examples. A database that can be audited (Tracy 2010) was maintained, and finally a detailed description of the setting and methods is provided (Tracy 2010).

### Ethical consideration

Permission from all the relevant hospital managers and the department of Health in the Eastern Cape was obtained before the study commenced. The study was registered with the Health Research Ethics Committee at Stellenbosch University (S14/10/240). Participation was voluntary and no data were collected before written informed consent was obtained. Informed consent included consent to audio record the focus group discussions.

## Results

Table 2 shows that the majority of participants were employed at Frere hospital.

**TABLE 2:** Background information on study participants.

Variables	Occupational therapists	Physiotherapists	Prosthetists
*N*	10	12	6
Gender	All women	10 women 2 men	All men
Age range	23–34 years	22–42 years	27–56 years
Employed at:			
Frere	8	6	6
Cecilia Makiwane	1	3	0
Empilweni	1	2	0
S.S. Gida	0	1	0

*Source*: Authors’ own work

Generally, the tool was received favourably by the participants as summarised by this opinion: ‘I think we should use it’. (Participant A, OT, FGD1). However, reservations were also voiced. Table 3 provides an overview of the themes that were identified during data analysis.

**TABLE 3:** Themes and subthemes identified from the findings.

Theme	Subthemes
Characteristics of the tool	Comprehensiveness Feasibility Format
A compass in patient management	Prosthetic prescription Managing the prosthetic waitlist Prosthetic preparation Patient education
Multidisciplinary nature	Nature of the tool Nature of the teams
Barriers to use	Time constraints Resistance to change
Application in the Eastern Cape province	-

*Source*: Authors’ own work

### Theme 1: Characteristics of the tool

#### Comprehensiveness

Participants from all three focus groups felt that the tool was comprehensive. The OTs had no reservations.

‘I found it very comprehensive and I was really happy to see that it accommodates for activities of daily living.’ (Participant A, OT, FGD1)

Some PTs agreed and one indicated that it might be more comprehensive than current assessments performed by them.

‘It’s nice that it elaborates more so it gives us a little more direction with the patients especially with their comorbidities; the medical aspects which we normally do not take into consideration.’ (Participant B, PT, FGD2)

Another PT had reservations and felt that the area of mobility is not subdivided into sufficient incremental steps.

‘It does not have an in between … there is just crutch walking and then wheelchair use. There is no mobilising with other assistive devices … maybe the person walks with a walking frame … there is no place to score … they could be very good on a walking frame, just lack balance. A lot of them (patients at the amputation clinic) are on a walking frame and mobilise very well on a walking frame, but then they have either fear or balance issues … so they do not want to progress to crutches then they do not really fit in there even though they can do a lot of the other stuff, they can do standing independently, throw and catch a ball, but they don`t fit in the walking section because there is only mobilising with crutches and they can`t walk with crutches.’ (Participant A, PT, FGD2)

#### Feasibility

Participants’ opinions on the user-friendliness of the tool varied. The consensus amongst OTs was. ‘It was easy to use, easy understandable’ (Participant A, OT, FGD1). Some prosthetists had reservations. ‘I understood the tool because it was explained, not easy to follow the tool if not explained’ (Participant F, P, FGD3).

#### Format

The same ambivalence was shown regarding the format of the tool.

‘Very simple to follow, user friendly, I don’t think it would give you any problems because it is quite self-explanatory.’ (Participant B, PT, FGD2)

The advantage of having the entire tool on one page was pointed out.

‘With the tool if everything is on one page it’s simple … so it’s not a burden.’ (Participant B, PT, FGD2)

A prosthetist had different views regarding the formatting of the tool.

‘Not easy to follow the format of the tool.’ (Participant F, P, FGD3)

### Theme 2: A compass in patient management

#### Prosthetic prescription

The prosthetists were sure that the tool could guide prosthetic prescription.

‘The tool is very useful because it give a picture on how to select a patient [*for prosthesis*].’ (Participant B, P, FGD3)

According to O&Ps, the tool fills an existing void.

‘We do not have guidelines to select patients [*for prosthesis*].’ (Participant F, P, FGD3)

While OTs felt that the tool could guide prosthetic prescription, they were uncertain where the final decision rests.

‘Who do you see as having the final say? Or does everyone have the right?’ (Participant E, OT, FGD1)

A colleague responded.

‘I do not know. Because, who would generally do the case management for something like this? I would think it would have to be a doctor.’ (Participant B, OT, FGD1)

#### Managing the prosthetic waitlist

Participants from all three focus groups agreed that using the tool might assist in addressing long waiting times: ‘the tool is a necessity to fight the backlog’ (Participant A, P, FGD3).

Prosthetists and PTs felt one way in which the tool can assist in dealing with the backlog was through prioritising.

‘You will be able to fast track patients who will benefit from things like this [*prosthesis*].’ (Participant B, PT, FGD2)

A PT argued that the tool might prevent the referral of people who are not prosthetic candidates.

‘The waiting periods will be a little bit adjusted … a lot of the time you end up with 80 something year-old patients … and then they are the ones jamming the [*wait*] list, because the doctors referred those patients for prosthesis but they don’t even fit the criteria, those patients might never use those things.’ (Participant B, PT, FGD2)

Use of the tool could also prevent prescription of prosthesis at an inappropriate time in the rehabilitation process.

‘So 32-year old trauma patient had a humerus fracture, femur fractures ended up with … stiff … knee joint from the femur fracture coz he never saw physio, but that doctor still sent that patient – he is in a wheelchair – to O&P to order prosthesis, because he thought the patient would need one without any other intervention. The patient can’t even stand, can’t walk.’ (Participant B, PT, FGD2)

A prosthetist summarises it:

‘The tool is very useful … because it eliminates unfairness to patients, so yes, the tool is needed.’ (Participant A, P, FGD3)

#### Prosthetic preparation

Participants pointed out that findings from the tool can be used to identify areas that must be addressed in order to prepare the patient for a prosthesis.

‘I think what is really nice about this tool is that if the patient … would not at the moment be able to get a prosthesis that there is a plan of action a way forward for them.’ (Participant B, OT, FGD1)

Another OT explained further.

‘If you see that this patient can be a good candidate but this patient has uncontrolled diabetes. So instead of sending the patient for prosthesis, to say: ‘I got a good score for this patient but the diabetes is poorly managed’. So you then take your patient to the doctor; discuss your findings with the doctor, presenting the tool and the doctor says: “OK fine we will help manage the diabetes. Really it encourages the team”.’ (Participant F, OT, FGD1)

#### Patient education

Tying in with prosthetic preparation was the issue of patient education.

‘I think it can be very educational to the patients, so that they can understand this is why you are not a candidate now. Let us work on these aspects and then they can take responsibility for their own rehab as well. Just yesterday, I issued a wheelchair to a patient … he ask me about the legs, prosthetic legs, and he is like, Those people say I am not ready, I can`t yet. He did not know why he is not ready.’ (Participant A, OT, FGD1)

### Theme 3: Multidisciplinary nature

#### The nature of the tool

The focus group participants agreed that amputee care requires a multidisciplinary approach and that this must be facilitated within the team. A role they thought the tool could play.

‘If all of us can work together to see the patient as a whole be it OT, physio, doctor, we all come up with one understanding, we have one criteria, we all follow the same set of rules … there is no blurred lines, no miscommunication. … It [*the tool*] addresses things like that.’ (Participant B, PT, FGD2)

The overall view from the participants was that the tool promotes collaboration amongst team members.

‘I thought it was nice that it was multidisciplinary just recognising that. I like it that way.’ (Participant B, OT, FGD1)

‘I think it encourages great team work.’ (Participant A, OT, FGD1)

#### The nature of the teams

In as much as the participants agreed on the multidisciplinary nature of the tool and the importance of teamwork, they did identify challenges in that regard which might negatively impact the use of the tool. It seems as if various professional groups operated independently rather than in teams.

‘We don’t have an understanding amongst all of us … the doctors are ordering, we [*PT*] are ordering, OTs are ordering, you guys are ordering [*Prosthetists*] … no communication across all … a patient that was an amp, specific patient, where the doctor had gone over and above and had just ordered the patient a prosthesis without taking anything (emphasises) into consideration, the patient was not mobilising, nothing! So he didn’t follow any criteria at all’ (Participant B, PT, FGD2)

‘Even after it was explained to him in detail [*that the patient will struggle to use a prosthesis*] he doesn’t see it.’ (Participant B & D together, PT, FGD2)

The strong emphasis participants placed on individual professional roles might indicate a lack of role release that might hamper teamwork.

‘Clear distinction [*should be made in the tool*] to who should answer what questions e.g. mark where a doctor should fill, physio should fill etc.’ (Participant A, P, FGD3)

‘The community part, community integration, your housework, is there a self-care? Yes, that domestic activities, that is all occupational therapy, specific for us.’ (Participant A, OT, FGD1)

One OT suggested that the tool could be split into different sections:

‘There are some parts which are not really applicable for us. So if you split it up for like medical team people you fill in this part, OTs you fill in this part, physios this part.’ (Participant C, OT, FGD1)

A counter-argument was brought by another OT.

‘I do not think it needs to be split specifically, because everyone can add their bit to each part. But there are definitely like certain things that the doctors will have to fill out and things that we would fill out.’ (Participant B, OT, FGD1)

### Theme 4: Barriers to use

The current nature of the team and teamwork, as described above, might cause a barrier to the use of the tool. Other barriers identified were time constraints and resistance to change.

#### Time constraints

Some participants felt that it took too long to complete the tool.

‘The only thing that I can just imagine sometimes just with time management and the amount of patients we see I think sometimes it would be difficult to make such a comprehensive thing.’ (Participant B, OT, FGD1)

However, the opinion that completing the tool was time-consuming was not shared by all. ‘Takes short time’. (Participant D, P, FGD3)

#### Resistance to change

An OT thought that general resistance to change might be a challenge in getting everyone to accept and use the tool. ‘New things to implement are generally a struggle, is it not?’ (Participant C, OT, FGD1). A colleague argued that the advantages of using the tool might break down this resistance.

‘Maybe because it is new … it will take effort from people to fill it in initially, but then when they see it does work … it will be better for them.’ (Participant B, OT, FGD1)

### Theme 5: Application in the Eastern Cape

Participants felt that there is a place for using the tool in the Eastern Cape province.

‘Much needed guideline for our province.’ (Participant C, P, FGD3)

They would use it.

‘I would [*use the tool*] as it is. Definitely.’ (Participant C, OT, FGD2)

Others felt, some modification is needed.

‘The tool needs small instructions … e.g. explaining the meaning of the asterisks and other information … in order to understand it.’ (Participant C, P, FGD3)

They also believed that unity is needed for the tool to produce fruitful results.

‘The thing is it will have to be accepted across the board with everybody (emphasis) for it to be effective … it’s pointless if just one group use the form.’ (Participant B, PT, FGD2)

## Discussion

Generally, study participants indicated that they found the tool comprehensive and relatively easy to use. They would use the tool, but would like to make minor changes. The tool is on one page, an advantage pointed out by some. However, this makes for a densely covered page and a need to follow the rows carefully to ensure that all aspects are completed and scored. The comment that the asterisks used in the tool must be explained showed this challenge. They are actually explained, but briefly. Thus, people who are unfamiliar with the tool might misunderstand or fail to see the explanation.

Study participants felt that patients on the waiting list could be screened with the tool in order to determine whether they are prosthetic candidates, need further pre-prosthetic rehabilitation, or would gain more from another rehabilitation strategy such as wheelchair mobility. However, there is a need for caution. The backlog dates back as far as 2008 and patients who have waited for a longer period might be disadvantaged because of weaknesses that had developed post-amputation. De Boer-Wilzing et al. (2011) and Roffman et al. (2014) showed that long prosthetic waiting times negatively affect prosthetic use and function. In fairness, these people should be offered an opportunity to recover lost strength and rebuild capacity (Fiedler et al. 2014) after which the tool can be completed again.

In addition to assisting with prosthetic prescription, participants felt that the tool could contribute to patient management in general. Some participants indicated that goals, referral and other management strategies could be based on the tool. While the tool can serve this purpose, it was not developed with a comprehensive assessment in mind. Many areas that one would cover in a comprehensive assessment are either not dealt with or dealt with superficially. It is in patients’ interests to use the tool for its specific purpose, that is, to determine prosthetic candidates or to identify areas that need to be addressed before prosthetic prescription, rather than as an overall assessment.

In the clinical opinion of some participants, the tool places too much emphasis on crutch walking to the detriment of people mobilising with walkers or rollators. However, crutch walking requires better balance on the remaining leg than walking with a walking frame or rollator. The ability to balance on the remaining leg is essential for functional walking with a prosthesis (Raya et al. 2010; Schoppen et al. 2003). Thus, it might be unwise to adjust the tool based on clinical opinion. Further evidence in this regard must be sought. In addition, should the person experience little trouble with the other aspects, the overall score will indicate that the person is potentially a good prosthetic candidate.

In accordance with findings from international (Gailey 2006; Schaffalitzky et al. 2011; Van Der Linde et al. 2003) and another South African study (Fredericks & Visagie 2013), these findings showed that prosthetic prescription was based on the clinical opinion of service providers. This leaves the process open to inconsistencies and possible unfairness to patients (Schaffalitzky 2010; Van der Linde et al. 2003). In this study setting, persons with amputations are managed in general surgical wards or as outpatients, rather than by teams dedicated to the care of amputees, as recommended by international guidelines (Geertzen et al. 2015). This, in conjunction with the information from the surgeon that doctors rotate frequently, leaves one with the concern that not everyone who prescribes prostheses has sufficient experience in the management and rehabilitation of persons with amputations to provide them with the expertise needed to do prescriptions.

Findings showed instances of prosthetic prescription where the cause of amputation, comorbidities, age and general physical condition were seemingly not taken into consideration. Evidence on the negative effect of these variables on a person’s ability to function with a prosthesis is clear from these studies (Davie-Smith et al. 2017; Mundell et al. 2016; Resnik & Borgia 2015; Roffman et al. 2014; Schoppen et al. 2003; Yilmaz et al. 2016). The tool, which takes these factors into consideration, might provide valuable guidance in this regard.

Our findings are similar to Ennion and Rhoda (2016), a situation where teamwork was lacking, and communication between various professional groups was limited. Prostheses were prescribed with seemingly little or no consultation between members of the multidisciplinary team. International guidelines in amputee management emphasise the importance of teamwork in amputee care, prosthetic prescription and rehabilitation (Geertzen et al. 2015).

Participants also indicated that they could not complete certain sections of the tool, which could traditionally be seen as the role of another professional group. While the various aspects in the tool cover the terrain of several professional groups, the tool does not require in-depth information on any aspect. Every member of the team should be able to ask the patient the necessary questions, do the quick tests and observe the stump to collect the information needed to complete the tool.

Concerns about who should calculate the final score or make the actual decision on prosthetic prescription were raised. The tool provides no specific directive in this regard. Practical decisions on the completion of the tool, calculation of scores and final decisions should be based on the specific circumstances and needs of individual teams and settings. If the tool is to be implemented successfully in this or any other setting, it is essential that all professional groups base prosthetic prescription on the tool. If used by only some, it will cause inconsistency in prescription, which, in turn, might lead to unfairness and inequity in patient management.

## Limitations

Not all participants had the opportunity to use the tool with patients prior to the focus group discussions. It was unfortunate that the orthopaedic surgeons chose not to participate in the study, and that general and vascular surgeons were not approached to participate, as the findings showed that doctors *do* prescribe prostheses in this study setting.

## Conclusion

These findings showed that the participants felt that this tool provided relevant, useful information and that it is easy to administer, but some felt that using it might be too time-consuming. The findings showed the haphazard manner in which decisions regarding prostheses are currently made in this setting, and the lack of teamwork. The tool may assist with these challenges and might be useful to manage the backlog for prostheses and to guide future prosthetic prescription in the Eastern Cape province. However, before implementation, further study on the tool is required and challenges in teamwork must be addressed. The teams should also develop strategies to deal with practical issues such as how they will complete the tool and come to a final decision.

## Recommendations

- It is recommended that the provincial government of the Eastern Cape implement evidence-based guidelines to guide prosthetic prescription. While these findings suggest that the tool might be able to serve as such a guideline, further study over a longer period of time and in different settings is required.
- It is recommended that the tool is further validated and tested for reliability, sensitivity and specificity.

## Footnotes

1 To be referred to as ‘the tool’ in the text.

## APPENDIX 1

**TABLE A1:** The layout and contents of the tool.

Aspect or standard	Contraindication† or poor prognosis	Negative predictor	Positive predictor
Aetiology	Any acutely terminal condition	Vascular or other progressive condition	Traumatic, congenital, orthopaedic or non-progressive condition
Number and level of amputations	Bilateral above knee amputations (AKA) in adults	Above and below knee or bilateral below knee	Unilateral above or below knee
Substance abuse including smoking	Continues with habit post-amputation	Has recently (2 years) stopped or cut down	No substance abuse in past 2 years
Ischaemic heart disease. ECG recommended	†Below knee amputation (BKA): Uncontrolled ischaemic heart disease (IHD); AK or bilateral amputation: even if good compliance and controlled	Good compliance and controlled (unilateral BKAs only)	No IHD
Cardiac failure (CF)	†Uncontrolled	Good compliance and controlled	No CF
Diabetes (DM)	Uncontrolled	Good compliance and controlled	No DM
Hypertension (HTN)	Uncontrolled	Good compliance and controlled	No HTN
Respiratory conditions	Uncontrolled	Good compliance and controlled	No past or current history
Body mass index	Underweight	Overweight	Within normal range
Continence	Incontinent bladder and bowel because of neurogenic causes	Other causes of incontinence	No bladder or bowel problems
Cognition (examine for stroke, head injury, multi-infarct dementia)	†Poor insight, judgement and reasoning; require supervision in daily activities	Limitations present but do not impact on activities of daily living	No cognitive fallout
Expectations	Unrealistic expectations of prosthesis, request for cosmetic prosthesis	Intermediate. Patient has not considered or is unaware of functional aspects of rehabilitation	Realistic expectations of prosthesis and role it has to play in complete rehab plan
Coordination and mobility with crutches (Can walk 200 m with crutches)	†Cannot mobilise with elbow crutches	Achieves basic standard only. Reasons for poor function are to be addressed	Unlimited mobility with crutches and can negotiate all terrains including steps
Wheelchair use	†Only uses wheelchair	Uses wheelchair for community access or when bilateral hand function is required	None
Stand on one leg independently; throw and catch a ball five times, hop and perform functional activities	Cannot	Achieves basic standard only. Reasons for poor function are to be addressed	Achieves standard with ease
Stand on remaining limb for 4 min	Cannot	Achieves basic standard only. Reasons for poor function are to be addressed	Achieves standard with ease
Stand up from sitting without using hands	†Cannot	Achieves basic standard only. Reasons for poor function are to be addressed	Achieves standard with ease
Self-care	†Dependent	Any degree of dependence	Totally independent
Domestic activities	Dependent	Any degree of dependence	Totally independent
Community activity: pre-morbid and current	†Bed bound	Active in house	Active in community
Remaining limb	Threatened	Questionable viability or deterioration in last 6 months	No problems. Good pulses and circulation
Amputation stump: range	†Fixed flexion deformity of hip and/or knee	Reduction in full range of hip and/or knee still to be addressed	Full range of movement with hip extension beyond neutral
Amputation stump: power	† < 4/5 hip extensors and abductors (BKA and AKA) and knee extensors (BKA). Patient generally weak	Good general strength < 4/5 hip extensors and abductors and knee extensors (BKA). Shortcomings to be addressed	5/5 all movements of hip and/or knee
Amputation stump: length AKA: 1/3 of opposite femur BKA: >12 cm–15 cm from knee joint line	Markedly shorter with minimal fulcrum	Shorter than standard	Meets standard or is longer
Amputation stump shape and soft tissue	Poor compliance or response with coning and mobilisation of soft tissues. Persistent dog ears and hard spots	Improvement in shape evident or anticipated. Surgical intervention considered	Conical form
Amputation stump: bony prominence causing soft tissue tension	Uncorrectable	Amenable to coning or surgical correction	No bony protuberances
Amputation stump: Wound healing	†Open wound, draining sinus	Healed but immobile scar	Healed and mobile scar
Amputation stump: skin condition	Thin skin or easily abrades with compression bandage Skin graft on weight bearing area	Healed skin grafts	Healthy, supple and flexible skin with no skin grafts on stump
Amputation stump pain or sensation	†Ischaemia	Neuroma, hypersensitive stump. Phantom pain impacting on function	No pain. Phantom pain not impairing function
**Total score both pages**			
Block in which highest score (most ticks) is obtained	Patient is not a prosthetic candidate. Reassess if factors are remediable. Put care plans into place	Remediate correctable factors through medical and therapeutic interventions	Potentially good candidate

*Source*: Based on Provincial Government of the Western Cape (PGWC), 2010, *Guidelines for screening of prosthetic candidates: Lower limb*, Provincial circular H176 of 2010, PGWC, Cape Town

AK, above knee; AKA, above knee amputations; BKA, below knee amputation; CF, cardiac failure; DM, diabetes; ECG, electrocardiography; HTN, hypertension; IHD, ischaemic heart disease.

† This means the variable is a contraindication for prosthetic fit.

The bold text summarises the three outcome options after applying the tool.
